# Retraction Note to: *Hericium erinaceus* mycelium and its isolated erinacine A protection from MPTP-induced neurotoxicity through the ER stress, triggering an apoptosis cascade

**DOI:** 10.1186/s12967-021-02748-y

**Published:** 2021-02-15

**Authors:** Hsing-Chun Kuo, Chien-Chang Lu, Chien-Heng Shen, Shui-Yi Tung, Meng Chiao Hsieh, Ko-Chao Lee, Li-Ya Lee, Chin-Chu Chen, Chih-Chuan Teng, Wen-Shih Huang, Te-Chuan Chen, Kam-Fai Lee

**Affiliations:** 1 Department of Nursing, Chang Gung University of Science and Technology, Chiayi, Taiwan; 2 Research Center for Industry of Human Ecology, Chang Gung University of Science and Technology, Taoyuan, Taiwan; 3 Division of Colorectal Surgery, Department of Surgery, Chang Gung Memorial Hospital, Kaohsiung Medical Center, Chang Gung University College of Medicine, Kaohsiung, Taiwan; 4 Department of Hepato‑Gastroenterology, Chang Gung Memorial Hospital, Chiayi, Taiwan; 5 Chang Gung University College of Medicine, Taoyuan, Taiwan; 6 Division of Colon and Rectal Surgery, Department of Surgery, Chang Gung Memorial Hospital, Chiayi, Taiwan; 7 Grape King Biotechnology Inc, Zhong‑Li, Taiwan; 8 Division of Nephrology, Kaohsiung Chang Gung Memorial Hospital and Chang Gung University College of Medicine, Kaohsiung, Taiwan; 9 Department of Pathology, Chang Gung Memorial Hospital, Chiayi, Taiwan

## Retraction to: J Transl Med (2016) 14:7810.1186/s12967-016-0831-y

The Editor-in-Chief has retracted this article [1] because two of the authors, Chin-Chu Chen and Li-Ya Lee, did not fully declare their association with Grape King Bio, which produces extracts of Hericium erinaceum (Lion’s mane mushroom). This undeclared competing interest would have affected interpretations of the work and recommendations from peer reviewers. In addition to this, Western blot rows Fas (33 kDa) and IP:TRAF2 WB: IRE1α (120 kDa) in Fig. 6 appear to duplicate each other.

Chien-Chang Lu agrees to this retraction. Hsing Chun Kuo, Meng Chiao Hsieh, Li-Ya Lee and Chin-Chu Chen do not agree to this retraction. Chien-Heng Shen, Shui-Yi Tung, Ko-Chao Lee, Chih-Chuan Teng, Wen-Shih Huang, Te-Chuan Chen and Kam-Fai Lee have not responded to any correspondence from the publisher about this retraction.
